# Ranked Adjusted Rand: integrating distance and partition information in a measure of clustering agreement

**DOI:** 10.1186/1471-2105-8-44

**Published:** 2007-02-07

**Authors:** Francisco R Pinto, João A Carriço, Mário Ramirez, Jonas S Almeida

**Affiliations:** 1 Instituto de Microbiologia, Instituto de Medicina Molecular, Faculdade de Medicina de Lisboa, Av. Professor Egas Moniz, 1649-028 Lisboa, Portugal; 2 Grupo de Biomatemática, Instituto de Tecnologia Química e Biológica, R. Quinta Grande, 6, 2780 Oeiras, Portugal; 3 Instituto de Engenharia de Sistemas e Computadores: Investigação e Desenvolvimento (INESC-ID), R. Alves Redol 9, 1000-029 Lisboa, Portugal; 4 Department of Biostatistics, and Applied Mathematics, Univ. Texas, MDAnderson Cancer Center, Houston, Texas, USA

## Abstract

**Background:**

Biological information is commonly used to cluster or classify entities of interest such as genes, conditions, species or samples. However, different sources of data can be used to classify the same set of entities and methods allowing the comparison of the performance of two data sources or the determination of how well a given classification agrees with another are frequently needed, especially in the absence of a universally accepted "gold standard" classification.

**Results:**

Here, we describe a novel measure – the Ranked Adjusted Rand (*RAR*) index. *RAR *differs from existing methods by evaluating the extent of agreement between any two groupings, taking into account the intercluster distances. This characteristic is relevant to evaluate cases of pairs of entities grouped in the same cluster by one method and separated by another. The latter method may assign them to close neighbour clusters or, on the contrary, to clusters that are far apart from each other. *RAR *is applicable even when intercluster distance information is absent for both or one of the groupings. In the first case, *RAR *is equal to its predecessor, Adjusted Rand (*HA*) index. Artificially designed clusterings were used to demonstrate situations in which only *RAR *was able to detect differences in the grouping patterns. A study with larger simulated clusterings ensured that in realistic conditions, *RAR *is effectively integrating distance and partition information. The new method was applied to biological examples to compare 1) two microbial typing methods, 2) two gene regulatory network distances and 3) microarray gene expression data with pathway information. In the first application, one of the methods does not provide intercluster distances while the other originated a hierarchical clustering. *RAR *proved to be more sensitive than *HA *in the choice of a threshold for defining clusters in the hierarchical method that maximizes agreement between the results of both methods.

**Conclusion:**

*RAR *has its major advantage in combining cluster distance and partition information, while the previously available methods used only the latter. *RAR *should be used in the research problems were *HA *was previously used, because in the absence of inter cluster distance effects it is an equally effective measure, and in the presence of distance effects it is a more complete one.

## Background

Grouping individual entities into sets with identical properties is a recurrent task in bioinformatics, taxonomy and phylogeny studies. When there are *a priori *reasons that allow the identification of properties that define each group, it is possible to use classification algorithms to distribute individuals among the possible classes. In other situations, different classes are defined without the absolute knowledge of what properties (and values of those properties) could identify "natural" classes. The usual procedure is the collection of data characterizing each individual, relate every pair of individuals through a distance measure computed from the data and perform clustering algorithms to find a "natural" grouping structure of those individuals based on the collected data. For simplicity, most of the remaining manuscript will use the term clustering, but the problems and methods presented are also applicable to classifications.

In some well established fields, researchers may assume a "gold standard" classification. If such gold standard is available, clustering results based on a particular kind of data can then be evaluated against it. False positives and false negatives can be identified and counted, enabling the computation of several related statistics. Even when gold standards are not available, different clusterings still need to be compared. Facing two different data sources characterizing the same set of biological entities and producing two different clusterings, one may wish to know to what extent and under which conditions one can maximize agreement or disagreement between two clusterings. This information may be useful to decide if it is worthwhile to collect and analyse both data sources since if their results are in complete agreement, then it may be enough to collect data from a single source. On the other hand, if the two clusterings disagree, combining their results may offer additional information and discriminatory power. Additionally, if the two data sources carry independent information, clusters that have a good match in both clusterings can be more reliable than clusters resulting from one data source alone.

### From previous measures to Ranked Adjusted Rand

Since the 70's researchers in statistics, psychology and biology, have developed methods to compare clusterings. If distance matrices between individual entities are available for both clusterings, it may be possible to directly correlate the pairwise distances [1]. But more frequently, researchers are interested in knowing if the resulting groups are similar or not. It is also possible to have highly correlated distance matrices that give rise to very different partitions due to scale heterogeneity in the distance values. Hence, the methods presented in the literature have been focused in the comparison of partitions (also designated flat clusterings), neglecting the closeness relationships between clusters. There are two main families of methods comparing partitions. One evaluating pairwise agreement (Rand, Adjusted Rand, Fowlkes-Mallows, Jaccard and Wallace indices) [2-6], the other searching for clusterwise agreement (Larsen, Meila's variation of information and Van Dongen indices) [7-9]. In both families, some methods are asymmetric, that is, the agreement of clustering *A *with *B *is different of the agreement of *B *with *A *[6,7]. This asymmetry can be helpful if the symmetric methods are being effected by the different discriminatory power of the two clusterings. Clusterwise methods are computed from a contingency table (*CT*, Table 1) that contains the dual classification of each individual entity in both clusterings, while pairwise methods are computed from a 2 by 2 mismatch matrix (*MM*, Table 2), derivable from the *CT*. Each of the four cells of *MM *count the pairs of entities that belong or not to the same cluster in either of the two clusterings. None of the two matrices *CT *or *MM *contain any information about the relatedness of the different clusters.

**Table 1:** Contingency Table (*CT*).

		*C'*				
			
		*C'*_1_	*C' *_2_	...	*C'*_*K'*_	*C *marginal totals
*C*	*C*_1_	*ct*_1,1_	*ct*_1,2_	...	*ct*_1,*K'*_	*n*_1_
	*C*_2_	*ct*_2,1_	*ct*_2,2_	...	*ct*_2,*K'*_	*n*_2_
	...	...	...	...	...	...
	*C*_*K*_	*ct*_*k*,1_	*ct*_*k*,2_	...	*ct*_*K*,*K'*_	*n*_*K*_
*C' *marginal totals		*n'*_1_	*n'*_2_	...	*n'*_*K'*_	*n*

Table used for the computation of cluster-wise measures of clustering agreement. *ct*_*ij *_is the number of entities that are both in cluster *C*_*i *_and *C'*_*j*_.

**Table 2:** Mismatch Matrix (*MM*).

		*c'*	
		
		Match	Mismatch
*c*	Match	*a*	*b*
	Mismatch	*c*	*d*

An auxiliary matrix for the computation of pair-wise measurements of clustering agreement. *a*, *b*, *c *and *d *represent counts of unique entity pairs.

Although the research in this area has produced many different methods, the classical methods are the most frequently referred, as an example, in a recent reference book on microarray data analysis, the only presented method to compare clusterings is equivalent to the Rand index [10]. On the other hand there is no general consensus on the choice of the method to compare clusterings, and active research on alternative methods was motivated by microarray and systems biology approaches [11]. It should be noted that the methods discussed here are not evaluating the quality or validating clustering algorithms. Instead the aim is to confront information of clusterings obtained from different data sources. It is also not a direct aim to achieve a combined better clustering closer to a hypothetical true classification. Nonetheless, these are possible secondary applications that are not tested in the present report. Additionally, researchers comparing clustering results should be aware that the measured levels of agreement could be strongly influenced by the inherent quality of the individual clusterings and by the type and quality of the datasets that originated the analysed clusterings.

The motivation to develop a new method stemmed from the observation that, for the available measures, when pairs of entities are in the same cluster on one clustering, and in different clusters on the other, it is considered irrelevant if these clusters are close neighbours or, on the contrary, very distant. A solution for such a problem was developed in a related subject, the quantification of the agreement of different observers performing a diagnostic test [12]. When the test has multiple possible categories with an ordinal relation (of disease severity, for example), weights are attributed to different degrees of disagreement. Minimal (when one observer chooses one category close to the one chosen by the other observer), intermediate and maximal disagreement (when the two observers choose categories in the two extremes of the ordinal scale), and these contribute proportionally to the overall measure of agreement computed.

The use of a similar weighting strategy in the comparison of clusterings is not directly applicable. First, and in contrast to the observer agreement case, the two clusterings are not forced to have the same number of groups. Second, there is no predetermined correspondence between the clusters in both clusterings. Third, the closeness relationships between clusters are frequently more complex (needing two or more dimensions to be correctly represented) than the simple ordinal scale of diagnostic categories (an unidimensional representation). The main achievements of the proposed measure are the solutions for these three problems. It consists on the definition of a new way to record pairwise agreements in a Ranked Mismatch Matrix (*RMM*), enabling the combined accounting of partition and intercluster distance information in the computation of an overall clustering agreement measure. The new measure was named Ranked Adjusted Rand (*RAR*), because it can be considered an expansion of the previous Hubert and Arabie (*HA*) adjusted Rand index and both measures are equivalent when there is no intercluster distance information available for both clusterings.

## Results and discussion

### Interpretation of *MDD *and *RAR *values

The Methods section describes how to compute *RAR *from a Ranked Mismatch Matrix (*RMM*, represented in Table 3) and the quantities Mean Diagonal Deviation (*MDD*) and expected *MDD *under independence of clusterings (*MDD*^*ind*^). *MDD *can be interpreted as the expected change in intercluster distance rank for a randomly chosen pair of entities. Considering one entity pair (*a*, *b*). If in clustering *C*, *b *belongs to the *r*^*th *^closest cluster to *a*'s cluster, then it is expected that in *C'*, *b *is in the *r *± (*MDD*^*ind *^× *K'*)^*th *^closest cluster to *a*'s cluster (*K' *is the number of clusters in *C'*). This kind of interpretation can be very useful if the aim is to predict the clusters obtained with one technique or data source using the clustering information obtained by a different technique or data source. *MDD *can take the value 1 only in a single situation: when in one clustering all entities are in the same clustering and in the other, every entity is in its own cluster, and all clusters are equally distant from each other. On the other hand, a *MDD *value of 0 corresponds to two clusterings with exactly identical partitions and equally ranked relative distances between clusters. The *RAR *values compare the observed *MDD *values with the theoretical *MDD *value if the assignment of entities to clusters was independent in both clusterings (the agreement in both clusterings would only be due to chance alone). The maximum value taken by *RAR *is 1, when *MDD *is 0. If *MDD *<*MDD*^*ind*^, the average entity pair tends to have smaller intercluster distance rank changes from one clustering to the other than it would have in the independence situation. In this case *RAR *takes positive values, meaning that the clusterings are more similar than expected by chance agreement. If *MDD *> *MDD*^*ind*^, *RAR *takes negative values, meaning that the deviation from perfect agreement is greater than expected by chance. The two last situations imply a very similar interpretation to the *HA *adjusted Rand case. *RAR *values are certainly less intuitive than interpreting simple Rand, but *RAR *provides more rich information about clustering agreement. *RAR *will be especially useful to distinguish situations in which *HA *or other measures are almost or completely identical. For these reasons we are proposing to use *RAR *in addition to previously available measures.

**Table 3:** Ranked Mismatch Matrix (*RMM*).

		*C'*				
		
		Match	Mismatch 1	Mismatch 2	...	Mismatch _*q*_
*C*	Match	*rmm*_1,1_	*rmm*_1,2_	*rmm*_1,3_	...	*rmm*_1,*q*+1_
	Mismatch 1	*rmm*_2,1_	*rmm*_2,2_	*rmm*_2,3_	...	*rmm*_2,*q*+1_
	Mismatch 2	*rmm*_3,1_	*rmm*_3,2_	*rmm*_3,3_	...	*rmm*_3,*q*+1_
	...	...	...	...	...	...
	Mismatch _*p*_	*rmm*_*p*+1,1_	*rmm*_*p*+1,2_	*rmm*_*p*+1,3_	...	*rmm*_*p*+1,*q*+1_

An auxiliary expanded matrix for the computation of pair-wise measurements of clustering agreement, accounting for intercluster distances. The mismatch row *i *indicates that the two entities are in different clusters *C*_*x *_and *C*_*y*_, and *C*_*y *_is the *i*^*th *^closest cluster to *C*_*x*_. Column meaning is analogous.

### *RAR *in the absence of intercluster distance information

When both clusterings being compared are flat, that is, when there is no intercluster distance information for any of them, two entities can only be either in the same cluster or in equally dissimilar clusters. *RMM *becomes identical to *MM*. In that situation, *1-MDD *is equal to the Rand index. Analogously, *RAR *becomes *HA*, since the correction for chance agreement is similar for both measures in the absence of any intercluster distance information. Proof of this equivalence is presented in Additional file 1.

### *RAR *with incomplete intercluster distance information

A major potential application of *RAR *is the comparison of a flat clustering with other for which interclusters distance information is available. One can use previously developed partition comparison measures to do this but the distance information available is neglected. However, *RAR *is able to compare both clusterings including the partial distance information available. The resulting *RMM *will have 2 × (*q*+1) (or (*p*+1) × 2) dimensions and it is possible to evaluate if the mismatches of the flat clustering tend to originate mismatches with larger rank differences in the other clustering than the flat matches.

This and the previous sections discussed the use of *RAR *when there is a partial or total absence of distance information. However, in most of the situations that Rand or Adjusted Rand indexes have been used, information about distance was indeed available. For an example see reference [11]. This is the most frequent situation when the partitions compared where produced by clustering algorithms. The clustering algorithm needs an inter-entity distance matrix, and this matrix is sufficient to derive the intercluster distances used for *RAR *computation. When the partitions are defined by classification methods, it may still be possible to have distance information, depending on the properties used to classify entities.

### *RAR *with ties in intercluster distances

A positive feature of the *R*(*i*, *j*) is that it is unnecessary to define rules to deal with rank ties. If a cluster has two neighbour clusters at the same distance, they will have the same intercluster distance rank. The only consequence is that the maximum intercluster distance rank (*p*+1 or *q*+1) decreases with the number of ties. *RMM *will have *p*+1 = *K *rows and *q*+1 = *K' *columns in the absence of ties in intercluster distance ranks. Each tie in *C *will reduce one row and each tie in *C' *will reduce one column to *RMM*. A higher number of ties can be due to a more discrete intercluster distance function and will produce a reduced *RMM *that is more similar to *MM*. The existence of ties is then responsible for the approximation of *RAR *to *HA*. This is consistent with the fact that more ties are a consequence of a lower resolution of the metric used to define the intercluster distance function. The minimal resolution corresponds to a binary distance function that can be 0 (same cluster) or 1 (different cluster) – that is, when *RAR *is equal to *HA*, as discussed previously.

### Design of small scale examples

**Figure 1:**
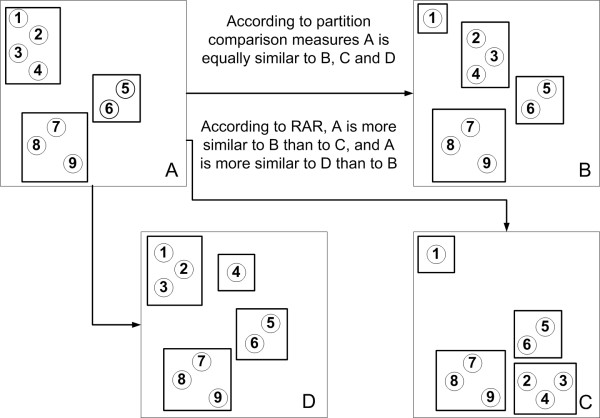
**Small clusterings example of *RAR*'s unique properties**. Clustering *A *divides 9 points (numbered circles) in three clusters identified by rectangles. By splitting the {1, 2, 3, 4} cluster, the clusterings *B*, *C *and *D *were formed. One of the child clusters kept the same location. The second child cluster moved away from the original location. In *B *and *C*, the second child cluster has only one entity, while in *D *it has three. In *B *and *D *the two split clusters are nearest neighbours, while in *C *they are maximally separated. The two dimensional coordinates of the points in the figure were used to compute average distances between clusters and to calculate *RAR *and other clustering comparison measures. The results are presented in Table 4.

To clearly show the desirable properties of the *RAR *measure compared with previously available methods, four theoretical simple clusterings were created (Figure 1). One of the four, clustering *A*, is the original one, with 9 entities divided in 3 clusters. The position of the points in each clustering is relevant. Two points that are more distant are less similar. Clusterings *B *to *D *were originated from *A *by splitting the {1, 2, 3, 4} cluster in two. One of the resulting clusters kept the same location, while the other varies in size and location in the different clusterings. The coordinates and cluster identity of every corresponding nine entities in the four clusterings were used to compute *RAR *and other ten measures of clustering agreement between *A *and each of its transformed clusterings. The description and formulas of these additional measures are available in the corresponding references given in Table 4, together with the values computed for these examples.

**Table 4:** Comparison of *RAR *with other measures applied to the small example of Figure 1.

	Clusterings compared		
Clustering comparison measures [reference]	*A-B*	*A-C*	*A-D*

Rand [2]	0.92	0.92	0.92
*HA *[4]	0.77	0.77	0.77
Jaccard [5]	0.70	0.70	0.70
Wallace forward [6]	0.70	0.70	0.70
Wallace reverse [6]	1.00	1.00	1.00
Larsen forward [7]	0.95	0.95	0.95
Larsen reverse [7]	0.81	0.81	0.81
*MH *[8]	0.89	0.89	0.89
Variation of Information [8]	0.11	0.11	0.11
Van Dongen [9]	1.00	1.00	1.00
***RAR***	**0.38**	**0.29**	**0.67**

Coordinates of the points in the four clusterings (*A*, *B*, *C *and *D*) of Figure 1 were used to compute *RAR *and other 10 measures of clustering agreement.

### Analysis of the small scale examples

The first point that these theoretical examples demonstrate is that *RAR*, contrary to the previous partition comparison measures, is able to detect a greater disagreement between clusterings if the entities causing the disagreement, besides changing the composition of the clusters change also the proximity relationships between clusters. That is shown by the difference in *RAR *value for the comparisons of *A *with *B *and *A *with *C *(Figure 1). All the other ten comparison measures consider *B *and *C *equally similar to *A*. In fact, the change in cluster composition from *A *to *B *is identical to the change from *A *to *C*. The difference is that the newly formed clusters in *B *are the closest neighbours, while in *C *they are the most distant clusters. As the discussed clusterings involve a small number of entities and clusters, and considering that the distance between points is proportional to the dissimilarity that was used to generate the clusterings, observation of Figure 1 clearly indicates that clustering *B *is more similar to *A *than *C *is.

On the contrary, *RAR *indicates that clustering *D *is more similar to *A *than *B *is. This arises because in *D *only one entity has a different location comparing with *A*. From *A *to *B *three entities changed position, although to the same relative location of the one entity cluster in *D*. Again, only *RAR *detected this difference, while all the other measures remained unchanged. This happens because *RAR *uses the intercluster distance ranks. The *RMM *comparing *A *with *B *will have more point pairs out of the diagonal than the comparison of *A *with *D*. In the first comparison three points changed their relative position, affecting the *RMM *position of 3 × 6 pairs of points. In the second comparison, only one point moved, hence, only 1 × 8 pairs of points can have different intercluster distance ranks than they would in a perfect match comparison. Consequently, *RAR *attributes more weight to the change from *A *to *B *than from *A *to *D*. From the point of view of partition comparison measures, *B *and *D *have equal differences relatively to *A*. They both result from *A *by splitting a cluster of 4 entities into one with 3 and other with 1 entity alone. This is the reason why the 10 partition comparison measures in Table 4 are not able to distinguish between the similarity of *A *with *B *and of *A *with *D*.

### Simulation of large scale clusterings

The two small comparisons of the previous section are extreme cases where the advantages of *RAR *were demonstrated, since it was able to detect differences between clusterings that none of the previously available methods where able to detect. But in realistic data sets, it is expected that a variable number of entities change their cluster membership and their relative position simultaneously. Additionally, some entity changes may contribute to make clusterings more similar while others may differentiate them. For these reasons, larger clusterings where simulated, also with more extensive entity shuffling. A complete description of these simulations is provided in Additional file 2. Briefly, five factors were systematically varied in simulated clustering comparisons: 1) number of entities, 2) number of clusters, 3) cluster size distribution, 4) fraction of entities changing cluster membership and relative position and 5) extension of change in relative position. The only factor that had an effect on the final *RAR *values was the fraction of entities changing cluster membership and location, producing a linear correlation coefficient of *r *= -0.918. The number of entities (*r *= 0.077), number of clusters (*r *= -0.109), and the cluster size distribution (*r *= -0.032) had negligible impact on *RAR *values. These low correlations support the conclusion that *RAR *values are not systematically influenced neither by the number of entities and clusters being compared nor by the distribution of cluster sizes. As the entities changing cluster membership were randomly selected from every possible cluster, the change in relative position of some entities could be balanced by entities moving in the opposite direction. Consequently, varying the extension of change in relative position produced highly variable results and a low correlation with *RAR *values (*r *= -0.016). To evaluate more precisely the influence of this factor on *RAR *values, a partial correlation analysis was performed on the relation between *RAR *values, *HA *values (that can be interpreted as *RAR *values without intercluster distance information) and the net change in entity relative position (measured by the correlation coefficient between the distance matrices of the two clusterings being compared). The results, presented in detail on supplementary material, show that *RAR *integrates independent information contained in the *HA *Index and in the correlation coefficient between distance matrices. The partial correlation of *RAR *with both factors are strong and positive (0.758 and 0.720), which means that both a higher fraction of entities changing cluster membership and a higher net change in entity relative position independently induce higher *RAR *values.

### Biological examples

To substantiate the general applicability of *RAR*, three examples with biological data are presented that compare 1) two microbial typing methods, 2) two gene regulatory network distances and 3) microarray gene expression data with pathway information. The first example is presented in the main text while the other two are included in Additional file 3.

Typing methods are major tools for the epidemiological characterization of bacterial pathogens, allowing the determination of clonal relationships between isolates based on their genotypic or phenotypic characteristics. Since typing schemes analyze different phenotypic or genotypic properties of bacteria, if some congruence between the methods is found, it suggests that a phylogenetic signal is being recovered by both methods, allowing greater confidence about evolutionary hypothesis or clonal dispersion of the strains under study. The same collection of bacterial isolates can be typed by different methodologies and it becomes of great epidemiological and evolutionary importance to understand the relationships between the clusters of isolates defined by the different methods. To this end we have recently evaluated the usefulness of a set of measures to quantitatively describe these relationships [13].

### Data handling

We analyzed the data generated by the characterization of a collection of 325 macrolide-resistant *Streptococcus pyogenes *[14,15]. This collection was characterized by *emm *sequencing, that generates groups of isolates differing by less than 92% in their DNA sequence and by comparison of the patterns generated after digestion of total DNA with the *SmaI *endonuclease and separation by pulsed-field gel electrophoresis (PFGE). Dice coefficient was used to compute dissimilarity between PFGE band patterns, enabling the subsequent hierarchical clustering with average linkage. Measurement of the agreement between the *emm *classification and PFGE clusterings for the same data set has already been done using *HA *and Wallace indices (W) [13]. Wallace index of clustering *A *relatively to *B *is the probability that two entities are in the same cluster in *B*, knowing they were in the same cluster in *A*. It is a pairwise asymmetric clustering agreement measure [6]. For the present work, PFGE clusterings were produced for 70 different Dice dissimilarity thresholds, covering all the possible values. Each of these clusterings was compared with the *emm *classification through *RAR*, *HA *and Wallace indices.

### Practical example results and discussion

**Figure 2:**
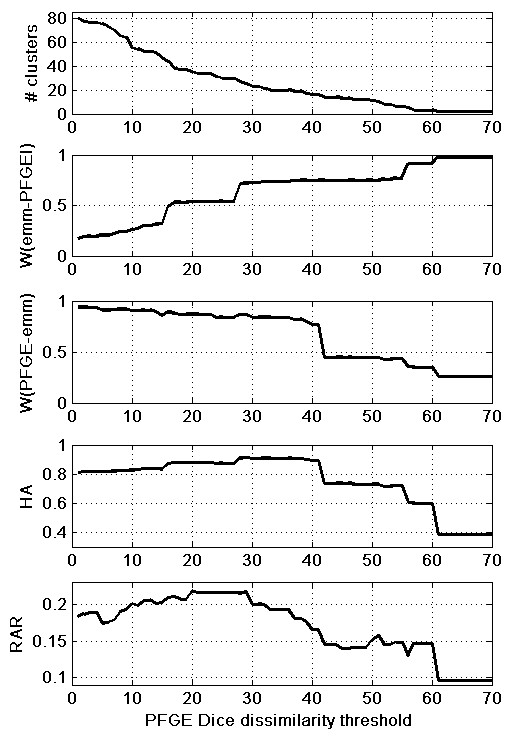
**Ranked Adjusted Rand (*RAR*), Adjusted Rand (*HA*) and Wallace (*W*) indices for the comparison of *emm *type with PFGE clusterings using different Dice dissimilarity thresholds**. Dice dissimilarity is in a 0–100 scale. The plot in the top indicates the number of PFGE clusters originated with the respective threshold, while the number of *emm *types is always 12. The minimum threshold studied, 1, does not originate 325 clusters because there are sets of isolates whose PFGE band patterns have a Dice dissimilarity of 0. *W*(*emm-PFGE*) is the probability that a pair of isolates is in the same PFGE cluster knowing that they have the same *emm *type. Analogously, *W*(*PFGE-emm*) is the probability that a pair of isolates has the same *emm *type knowing that they are in the same PFGE cluster. *HA *reflects the evolution of both Wallace indices. The plateau of maximum *HA*, between the thresholds of 28 and 41, is a region of compromise where both Wallace indices are high. The curve of *RAR *values shows a more complex behaviour, with a plateau of maximum values between the thresholds of 20 and 29, and a significant decrease between 29 and 41, where *HA *is nearly constant.

The dendrogram built with PFGE and *emm *type classification data is shown in Figure 1 of the Additional file 3. Although major agreements for half of the *emm *types (1,4, 9, 11, 12 e 22) are identifiable within the dendrogram, it becomes a hard task to quantify the overall concordance including the other types. In order to compare the *emm *classification and the clustering with PFGE it is first required to define the threshold that maximizes the agreement for the two microbial typing methods. Figure 2 shows the values of *RAR*, *HA *and Wallace indices for the range of Dice dissimilarity thresholds used on the PFGE clustering.

#### Wallace index

As the threshold increases, the number of PFGE clusters diminishes, resulting on a set of larger clusters. On this process, the Wallace index of *emm *classification relative to PFGE increases, meaning that the probability that two isolates are grouped in the same PFGE cluster if they share the same *emm *type increases. Also, the fact that PFGE clusters are larger raises the probability that any two isolates belong to the same cluster. The step like increases on the ascending curve corresponds to the collapse of clusters that had many isolates with the same *emm *type. The Wallace index of PFGE relative to *emm *type, which matches the probability that two isolates have the same *emm *type, knowing that they are on the same PFGE cluster, shows the opposite behaviour. On this case, the step like decreases on the curve correspond to the collapse of clusters rich in different *emm *types.

#### HA index

The *HA *curve reflects a compromise of the patterns of the two Wallace index curves, with a maximum around a 29% Dice similarity threshold, where both Wallace index curves present simultaneously relatively high values. *HA *is therefore similar to an average of the two Wallace indices, corrected by chance agreement.

#### RAR

**Figure 3:**
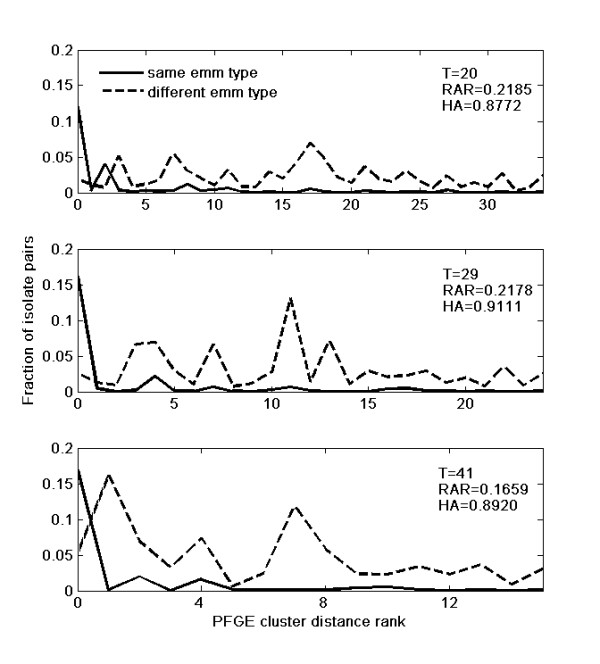
**Ranked Mismatch Matrix (*RMM*) composition at different Dice dissimilarity thresholds for PFGE clustering**. The *RMMs *for the comparison of *emm *type with PFGE clusterings have dimensions *p *× 2, where *p *depends on the number of PFGE clusters and the two columns correspond to isolate pairs with the same or with different *emm *type. The PFGE intercluster distance rank is represented in the horizontal axis. The isolate pairs with the same *emm *type are represented with full lines while for pairs with different *emm *type a dashed line was used. The frequencies plotted in the vertical axis are relative, meaning that the content of each *RMM *element was divided by the sum of all *RMM *elements. It corresponds to the fraction of isolate pairs contributing for the respective *RMM *element. *RMM *composition was studied at three different thresholds (*T *= 21, 29 and 41) because, 21 is an optimal threshold for *RAR *but not for *HA*, 29 is an optimal threshold for both measures and 41 is a slightly sub-optimal threshold for *HA *(it is at the end of the maximal plateau of *HA *in Figure 3) and a bad threshold for *RAR*. The frequency distributions of isolate pairs with the same *emm *type are similar for the three thresholds. This is not the case for isolate pairs with different *emm *type. Here, as the threshold increases, the frequency peaks become larger and occur at lower cluster distance ranks, contributing in this way for a weaker agreement.

*RAR *shows a distinct behaviour from the other measures. In opposition to *HA *and Wallace indices, *RAR *variation is not dominated by large regions of no or low variability of the measure, meaning that *RAR *is sensitive to factors that are not influencing the other measures. The *RAR *curve presents two similar maxima for thresholds at 20% and 29% Dice dissimilarity, with values of 0.2185 and 0.2178 respectively. These two points limit a window where *RAR *is nearly constant. The *RAR *threshold at 29% corresponds to the maximum value of *HA*, 0.9111. On the *HA *curve, this point marks the beginning of a low-variation region between Dice dissimilarity thresholds of 28% and 41%. This window is actually where the two measures, *RAR *and *HA*, disagree the most: *HA *is nearly constant while *RAR *is decreasing considerably. To clarify this different behaviour, *RMM *compositions for thresholds 20, 29 and 41 are shown on Figure 3.

#### RMM analysis for specific thresholds

Due to the fact that the *emm *classification does not offer distances between the different types, two isolates can only be labelled as being of the same *emm *type or not. This being so, *RMM *holds two colums (one with the isolates pairs with the same *emm *type and another with the isolates pairs with different *emm *types) and a number of lines corresponding to the maximum value of the PFGE inter cluster distance rank for each threshold. Figure 3 shows three plots where each of these two columns is represented by a curve. On these plots, the frequencies of the isolates pairs are relative so that the sum of all the represented points, including both curves, is 1. For the three studied thresholds, the frequency distributions of isolate pairs with the same *emm *type for different cluster distance ranks are very similar. The major difference is that the plots for thresholds 29% and 41% show a higher frequency for isolate pairs with the same *emm *type and a cluster distance rank of 0, meaning that the pair is in the same PFGE cluster. It is this fact that is responsible for the higher values of *HA *for these thresholds. On the other hand, *HA *is not able to detect the differences in the frequency distribution of isolate pairs with different *emm *types for different cluster rank distances. Compared with thresholds 29% and 41%, this distribution for threshold 20% is flatter, meaning that isolate pairs with different *emm *types are more homogeneously distributed throughout the cluster distance rank scale. For the higher thresholds there are stronger peaks in this distribution, and they occur in the first half of the cluster distance rank scale. This contributes to a weaker agreement. For the threshold 29%, the increase in the frequency of pairs with the same *emm *type and in the same PFGE cluster balances the effect of the peaks in the distribution of pairs with different *emm *type, thus *RAR *is practically identical to the one for the threshold of 20%. For the threshold 41% the peaks are stronger, occur at lower values of cluster distance rank and there is no counteracting effect, causing a significant decrease in *RAR *value that is not observed for *HA*. In fact, to compute *HA *the frequencies of isolate pairs with different *emm *type and cluster distance rank greater than 1 are grouped in just one class. This is not the case for *RAR *that uses all the values in the *RMM *for its computation. One can argue that the higher peaks in the frequencies of isolate pairs for higher thresholds are due to the lower number of clusters. Fewer clusters correspond to fewer degrees of freedom in clustering formation. With more clusters it becomes easier to build clusterings with a more perfect agreement. As *RAR *computes a weighted average over all isolate pairs, in all *RMM *positions, it is more sensitive to the shape of the distribution of frequencies along the different matrix elements than to the actual frequency values. If for the different thresholds studied, the frequency distributions of isolate pairs for different cluster distance ranks were the same, *RAR *would give similar results to *HA*, which is expectable since *RAR *is an extension of the *HA *method. The main difference is that *RAR *is sensitive to changes in the discussed distributions, or, in other words, it is sensitive to different levels of disagreement when a pair of isolates is not in the same class or cluster. This practical example shows the power of using *RAR *jointly with clustering comparison measures that only evaluate partition divergence, like *HA *or the Wallace coefficients. Using *HA *alone, it would be difficult to choose a Dice dissimilarity threshold in the interval of 28 to 41%. Between those values the partitions compared are almost equally similar, and the gains in *W*(*emm-PFGE*) are compensated by lower *W*(*PFGE-emm*). But *RAR *values are clearly higher for the 29% than for the 41% threshold. As the *HA *partition similarity is practically the same for both thresholds, it is safe to say that the change in *RAR *is due to an intercluster distance disagreement effect. Comparing the variation of *RAR *values with the corresponding variation of *HA *or other measures provides an easier way to infer the meaning of *RAR *values. *RAR *has another maximum at 20% dissimilarity threshold, and the corresponding *HA *value is not in the maximal plateau. This means that looking only at partition information, a 20% threshold would be inferior to 28% or 41%, but at 20% the entity pairs have a stronger tendency to be in equally separated clusters in both clusterings, which increases the *RAR *value. The existence of a *RAR *maximum at this value is actually confirming the empirically accepted Dice dissimilarity threshold of 20% to define PFGE clusters [13,15-17], a value that does not correspond to the *HA *maximum.

## Conclusion

As previously stated, comparing different clusterings for the same set of entities is a recurrent task. Hubert and Arabie's Adjusted Rand (*HA*) index is still commonly used to quantify these comparisons [11,13,18,19]. The new method described here, the Ranked Adjusted Rand (*RAR*), can be useful in all instances where *HA *is applicable. *RAR *is an extension of *HA*, and produces identical results when there is no intercluster distance information. The novelty introduced by the *RAR *measure is the way the Ranked Mismatch Matrix (*RMM*) is built. The fact that the contribution of each entity pair in *RMM *is determined by the intercluster distance rank function allows the recording of different levels of disagreement circumventing the problem of pre-ordering clusters and of difference of number of clusters in both clusterings.

The artificial small examples highlighted the situations where *HA *and other available measures are not able to discriminate while *RAR *is. Namely, when from one clustering to another, a cluster is split in two and one or two of the child clusters change their localization relatively to the remaining clusters, only *RAR *is sensitive to differences in the relative distances of these new clusters as compared with the original clustering.

When applied to the comparison of larger clusterings, *RAR *proved to be robust to factors like number of entities and clusters, and also to different cluster density patterns. From the viewpoint of computation time needed to execute *RAR *no special problems are anticipated even with its application to very large clusterings. Simulated clustering comparisons clarified that the distance information that *RAR *integrates is not the same that is already implicit in the partition information. For constant partition information *RAR *is still sensitive to distance information changes. Analogously, for constant correlation between distance matrices, *RAR *is still sensitive to changes in partitions.

*RAR *was tested with experimental data from the field of molecular epidemiology. The test case was a comparison between one flat classification, without interclass distance information, the *emm *types, and a hierarchical clustering, from PFGE data, where there was inter cluster distance information for several clusterings originated from the same dendrogram. *RAR *produced different results from *HA *and Wallace indices. Analysis of *RMM *content proved to be helpful in the detection of what disagreements or agreements were causing changes in *RAR *and *HA *values. In conclusion, use of the *RAR *measure lead to a more informed decision on the best threshold to generate a PFGE clustering with a maximum agreement with the *emm *type classification. Although measures like Rand, Jaccard and Wallace indices continue to be useful, especially because the numbers generated have an associated intuitive meaning, we argue that *RAR *supersedes the previous indices when measuring the overlap between clusterings or classifications. The foundation of this argument lays in the fact that *RAR *is sensitive to the same partition differences that previous methods also detected, but in addition it is also sensitive to intercluster distance changes.

## Methods

### *RAR *description

A clustering *C *is a partition of the set of objects *D*, with *n *elements (identified below by the letters *i *and *j*), into sets (clusters) *C*_1_, *C*_2_,...*C*_*K*_, with *n*_1_, *n*_2_,...*n*_*K *_number of entities, all greater than 0. The task of measuring clustering agreement arises when, for the same set *D*, two different methods are used to produce two different clusterings, *C *and *C'*, with *K *and *K' *clusters each. To evaluate the overlap of the two partitions, a contingency table is built, where every element of *D *contributes to the cell of the corresponding clusters in both *C *and *C' *as shown in Table 1. Focusing on the pairwise agreement, the information in *CT *can be further condensed in a mismatch matrix represented in Table 2, where *a*, *b*, *c *and *d *represent the counts of entity pairs that fall in each of the four possible categories. For example, entity pairs in the *b *category are in the same cluster in *C *but in different clusters in *C'*. The sum of *a*, *b*, *c *and *d *is *n*(*n*-1)/2, the total number of unique entity pairs.

### Adjusted Rand, the *RAR *predecessor

Hubert and Arabie proposed an adjusted Rand index to quantify clustering agreement [4]:

HA(C,C′)=a+d−nca+b+c+d−nc     (1)
 MathType@MTEF@5@5@+=feaafiart1ev1aaatCvAUfKttLearuWrP9MDH5MBPbIqV92AaeXatLxBI9gBaebbnrfifHhDYfgasaacH8akY=wiFfYdH8Gipec8Eeeu0xXdbba9frFj0=OqFfea0dXdd9vqai=hGuQ8kuc9pgc9s8qqaq=dirpe0xb9q8qiLsFr0=vr0=vr0dc8meaabaqaciaacaGaaeqabaqabeGadaaakeaacqWGibascqWGbbqqcqGGOaakcqWGdbWqcqGGSaalcuWGdbWqgaqbaiabcMcaPiabg2da9maalaaabaGaemyyaeMaey4kaSIaemizaqMaeyOeI0IaemOBa42aaSbaaSqaaiabdogaJbqabaaakeaacqWGHbqycqGHRaWkcqWGIbGycqGHRaWkcqWGJbWycqGHRaWkcqWGKbazcqGHsislcqWGUbGBdaWgaaWcbaGaem4yamgabeaaaaGccaWLjaGaaCzcamaabmaabaGaeGymaedacaGLOaGaayzkaaaaaa@4B69@

Where *n*_*c *_is the correction for chance agreement, corresponding to the expected sum of *a *and *d *if *C *and *C' *where totally independent clusterings:

nc=n(n2+1)−(n+1)∑k=1Knk2−(n+1)∑k′=1K′n′k′2+2∑k=1K∑k′=1K′nk2n′k′2n2(n−1)     (2)
 MathType@MTEF@5@5@+=feaafiart1ev1aaatCvAUfKttLearuWrP9MDH5MBPbIqV92AaeXatLxBI9gBaebbnrfifHhDYfgasaacH8akY=wiFfYdH8Gipec8Eeeu0xXdbba9frFj0=OqFfea0dXdd9vqai=hGuQ8kuc9pgc9s8qqaq=dirpe0xb9q8qiLsFr0=vr0=vr0dc8meaabaqaciaacaGaaeqabaqabeGadaaakeaacqWGUbGBdaWgaaWcbaGaem4yamgabeaakiabg2da9maalaaabaGaemOBa4MaeiikaGIaemOBa42aaWbaaSqabeaacqaIYaGmaaGccqGHRaWkcqaIXaqmcqGGPaqkcqGHsislcqGGOaakcqWGUbGBcqGHRaWkcqaIXaqmcqGGPaqkdaaeWbqaaiabd6gaUnaaDaaaleaacqWGRbWAaeaacqaIYaGmaaGccqGHsislcqGGOaakcqWGUbGBcqGHRaWkcqaIXaqmcqGGPaqkdaaeWbqaaiqbd6gaUzaafaWaa0baaSqaaiqbdUgaRzaafaaabaGaeGOmaidaaOGaey4kaSIaeGOmaiZaaabCaeaadaaeWbqaamaalaaabaGaemOBa42aa0baaSqaaiabdUgaRbqaaiabikdaYaaakiqbd6gaUzaafaWaa0baaSqaaiqbdUgaRzaafaaabaGaeGOmaidaaaGcbaGaemOBa4gaaaWcbaGafm4AaSMbauaacqGH9aqpcqaIXaqmaeaacuWGlbWsgaqbaaqdcqGHris5aaWcbaGaem4AaSMaeyypa0JaeGymaedabaGaem4saSeaniabggHiLdaaleaacuWGRbWAgaqbaiabg2da9iabigdaXaqaaiqbdUealzaafaaaniabggHiLdaaleaacqWGRbWAcqGH9aqpcqaIXaqmaeaacqWGlbWsa0GaeyyeIuoaaOqaaiabikdaYiabcIcaOiabd6gaUjabgkHiTiabigdaXiabcMcaPaaacaWLjaGaaCzcamaabmaabaGaeGOmaidacaGLOaGaayzkaaaaaa@7BB3@

Milligan and Cooper [20] and more recently Steinley [21] performed comparative studies of several pairwise clustering agreement criteria. They found *HA *the criterion with the most desirable properties, especially the zero expected value in the case of independent clusterings and the robustness to changes in cluster number and cluster size heterogeneity. The basic principle of *HA *is to compute the fraction of entity pairs in the diagonal of *MM*, because those pairs are the ones contributing to the general agreement. The pairs in *b *and *c *have a null contribution to the agreement. This fraction must be corrected for the expected chance agreement.

### Ranked Mismatch Matrix (*RMM*), a new format for the presentation of clustering data

To include the intercluster distance information, the entity pairs in *a *should continue to have a maximum contribution to the overall agreement, but *b, c *and *d *entity pairs should have different contributions according to the degree of mismatch in each of the two clusterings. First an intercluster distance rank function *R *is defined for every pair of entities (*i*, *j*) of a data set *D *(expression 3).

*R*(*i*, *j*) = (*x*, *y*): *i*, *j *∈ {1,2,...*n*}; *x *∈ {1,2,...*K *- 1}; *y *∈ {1,2,...*K' *- 1}     (3)

*R*(*i*, *j*) = (*x*, *y*), means that in clustering *C*, entity *j *is in the *x*^*th *^cluster closer to the one of entity *i*, and in clustering *C'*, the cluster of entity *j *is the *y*^*th *^closer to the cluster of *i*. In the case *i *and *j *are in the same cluster in *C*, *x *will be 0. If *i *and *j *are in the same cluster in *C'*, *y *will be 0. The distance between two clusters is here measured as the average distance between their entities. This is only possible when distances between every pair of entities are available. According to the problem, other intercluster distance function can be defined. For instance the standard single, complete or other linkage functions of hierarchical clustering can be used. In the absence of any distance information, the distance between a cluster and itself is 0 and between two different clusters is 1. Additionally, the intercluster distance definition does not have to be the same in the two clusterings being compared. These definitions allow the *RAR *method to be applied to any pair of clusterings. With the help of the intercluster distance rank function the Ranked Mismatch Matrix (*RMM*), represented in Table 3, can be computed, with the general element *rmm*_*x*,*y *_defined as:

rmmx,y=∑i=1n∑j=1nH(i≠j)H(R(i,j)=(x−1,y−1))     (4)
 MathType@MTEF@5@5@+=feaafiart1ev1aaatCvAUfKttLearuWrP9MDH5MBPbIqV92AaeXatLxBI9gBaebbnrfifHhDYfgasaacH8akY=wiFfYdH8Gipec8Eeeu0xXdbba9frFj0=OqFfea0dXdd9vqai=hGuQ8kuc9pgc9s8qqaq=dirpe0xb9q8qiLsFr0=vr0=vr0dc8meaabaqaciaacaGaaeqabaqabeGadaaakeaacqWGYbGCcqWGTbqBcqWGTbqBdaWgaaWcbaGaemiEaGNaeiilaWIaemyEaKhabeaakiabg2da9maaqahabaWaaabCaeaacqWGibascqGGOaakcqWGPbqAcqGHGjsUcqWGQbGAcqGGPaqkcqWGibascqGGOaakcqWGsbGucqGGOaakcqWGPbqAcqGGSaalcqWGQbGAcqGGPaqkcqGH9aqpcqGGOaakcqWG4baEcqGHsislcqaIXaqmcqGGSaalcqWG5bqEcqGHsislcqaIXaqmcqGGPaqkcqGGPaqkaSqaaiabdQgaQjabg2da9iabigdaXaqaaiabd6gaUbqdcqGHris5aaWcbaGaemyAaKMaeyypa0JaeGymaedabaGaemOBa4ganiabggHiLdGccaWLjaGaaCzcamaabmaabaGaeGinaqdacaGLOaGaayzkaaaaaa@6280@

*H*(*x*) is a Heaviside step function that takes the value 1 when *x *is true and 0 otherwise. The double sum includes the equal entity pairs of type (*i*, *i*) and the repeated entity pairs of types (*i*, *j*) and (*j*, *i*). The pairs of the first type do not contribute to the final sum due to the Heaviside function *H*(*i*≠*j*). The repeated pairs (differing only by the order of the entities inside the pair) need to be accounted in the sum because, for each of the individual clusterings, the intercluster distance rank is not necessarily symmetric. As an example, cluster *A *may be the closest neighbour of cluster *B*, but the closest neighbour of cluster *B *may be *C *and not *A*. In *RMM*, intercluster distance rank information for every pair of entities is recorded without identifying which clusters are separated at what rank distance. This is important because for each cluster, the *i*^*th *^neighbour cluster can be different. In this way, the intercluster distance information can be integrated with the partition comparison without the need of a strict ordinal relationship between clusters (like the example of disease severity referred in the introduction), of a known cluster correspondence between clusterings or of an equal number of clusters in both clusterings.

### Measuring clustering agreement

For two very similar clusterings, the majority of the entity pairs would contribute for elements close to the matrix diagonal. Even if *RMM *is not square, an alternative geometrical diagonal can be traced, linking the centre of the *rmm*_1,1 _element (with coordinates (0,0)) with the center of the *rmm*_*p*+1,*q*+1 _element (with coordinates (*p*, *q*)) If, on the contrary, the clusterings disagree to a large extent, most entity pairs will be far from the diagonal, concentrated around *rmm*_*p*+1,1 _and *rmm*_1,*q*+1_. From these considerations it immediately follows that a good measure of cluster disagreement is the Mean Diagonal Deviation (*MDD*) for all the entity pairs in *RMM*.

MDD=∑i=1p+1∑j=1q+1rmmi,j⋅|i−1p−j−1q|n2−n     (5)
 MathType@MTEF@5@5@+=feaafiart1ev1aaatCvAUfKttLearuWrP9MDH5MBPbIqV92AaeXatLxBI9gBaebbnrfifHhDYfgasaacH8akY=wiFfYdH8Gipec8Eeeu0xXdbba9frFj0=OqFfea0dXdd9vqai=hGuQ8kuc9pgc9s8qqaq=dirpe0xb9q8qiLsFr0=vr0=vr0dc8meaabaqaciaacaGaaeqabaqabeGadaaakeaacqWGnbqtcqWGebarcqWGebarcqGH9aqpdaWcaaqaamaaqahabaWaaabCaeaacqWGYbGCcqWGTbqBcqWGTbqBdaWgaaWcbaGaemyAaKMaeiilaWIaemOAaOgabeaaaeaacqWGQbGAcqGH9aqpcqaIXaqmaeaacqWGXbqCcqGHRaWkcqaIXaqma0GaeyyeIuoakiabgwSixpaaemaabaWaaSaaaeaacqWGPbqAcqGHsislcqaIXaqmaeaacqWGWbaCaaGaeyOeI0YaaSaaaeaacqWGQbGAcqGHsislcqaIXaqmaeaacqWGXbqCaaaacaGLhWUaayjcSdaaleaacqWGPbqAcqGH9aqpcqaIXaqmaeaacqWGWbaCcqGHRaWkcqaIXaqma0GaeyyeIuoaaOqaaiabd6gaUnaaCaaaleqabaGaeGOmaidaaOGaeyOeI0IaemOBa4gaaiaaxMaacaWLjaWaaeWaaeaacqaI1aqnaiaawIcacaGLPaaaaaa@630B@

The quantity inside the modulus is the normalized distance of the element (*i*, *j*) to the *RMM *diagonal, such that for the more distant elements (*rmm*_1,*q*+1 _and *rmm*_*p*+1,1_) it takes the value of 1. Consequently, the maximum value of *MDD *is also 1. The modulus implies that *MDD *is always greater or equal to 0. To obtain a measure of agreement between clusterings it is enough to compute 1-*MDD*, although this quantity is not yet corrected for chance agreement. To perform this correction, the expected *MDD *value under independence of clusterings *C *and *C' *(conditional on the marginals of *CT *and on the intercluster ranked distances) must be known. To compute this *MDD*^*ind *^it is first necessary to build *RMM*^*ind *^according to:

rmmx,yind=(∑i=1K∑j=1K(H(R((∀s:s∈Ci),(∀t:t∈Cj))=(x−1,⋅))⋅ni⋅nj−H(i=j)⋅ni))×(∑i=1K′∑j=1K′(H(R((∀s:s∈Ci′),(∀t:t∈Cj′))=(⋅,y−1))⋅ni′⋅nj′−H(i=j)⋅ni′))/(n2−n)     (6)
 MathType@MTEF@5@5@+=feaafiart1ev1aaatCvAUfKttLearuWrP9MDH5MBPbIqV92AaeXatLxBI9gBaebbnrfifHhDYfgasaacH8akY=wiFfYdH8Gipec8Eeeu0xXdbba9frFj0=OqFfea0dXdd9vqai=hGuQ8kuc9pgc9s8qqaq=dirpe0xb9q8qiLsFr0=vr0=vr0dc8meaabaqaciaacaGaaeqabaqabeGadaaakeaafaqabeGabaaabaGaemOCaiNaemyBa0MaemyBa02aa0baaSqaaiabdIha4jabcYcaSiabdMha5bqaaiabdMgaPjabd6gaUjabdsgaKbaakiabg2da9maabmaabaWaaabCaeaadaaeWbqaaiabcIcaOiabdIeaijabcIcaOiabdkfasjabcIcaOiabcIcaOiabgcGiIiabdohaZjabcQda6iabdohaZjabgIGiolabdoeadnaaBaaaleaacqWGPbqAaeqaaOGaeiykaKIaeiilaWIaeiikaGIaeyiaIiIaemiDaqNaeiOoaOJaemiDaqNaeyicI4Saem4qam0aaSbaaSqaaiabdQgaQbqabaGccqGGPaqkcqGGPaqkcqGH9aqpcqGGOaakcqWG4baEcqGHsislcqaIXaqmcqGGSaalcqGHflY1cqGGPaqkcqGGPaqkcqGHflY1cqWGUbGBdaWgaaWcbaGaemyAaKgabeaakiabgwSixlabd6gaUnaaBaaaleaacqWGQbGAaeqaaOGaeyOeI0IaemisaGKaeiikaGIaemyAaKMaeyypa0JaemOAaOMaeiykaKIaeyyXICTaemOBa42aaSbaaSqaaiabdMgaPbqabaaabaGaemOAaOMaeyypa0JaeGymaedabaGaem4saSeaniabggHiLdaaleaacqWGPbqAcqGH9aqpcqaIXaqmaeaacqWGlbWsa0GaeyyeIuoakiabcMcaPaGaayjkaiaawMcaaiabgEna0cqaamaalyaabaWaaeWaaeaadaaeWbqaamaaqahabaGaeiikaGIaemisaGKaeiikaGIaemOuaiLaeiikaGIaeiikaGIaeyiaIiIaem4CamNaeiOoaOJaem4CamNaeyicI4Saem4qam0aaSbaaSqaaiabdMgaPbqabaaccaGccqWFYaIOcqGGPaqkcqGGSaalcqGGOaakcqGHaiIicqWG0baDcqGG6aGocqWG0baDcqGHiiIZcqWGdbWqdaWgaaWcbaGaemOAaOgabeaakiab=jdiIkabcMcaPiabcMcaPiabg2da9iabcIcaOiabgwSixlabcYcaSiabdMha5jabgkHiTiabigdaXiabcMcaPiabcMcaPiabgwSixlabd6gaUnaaBaaaleaacqWGPbqAaeqaaOGae8NmGiQaeyyXICTaemOBa42aaSbaaSqaaiabdQgaQbqabaGccqWFYaIOcqGHsislcqWGibascqGGOaakcqWGPbqAcqGH9aqpcqWGQbGAcqGGPaqkcqGHflY1cqWGUbGBdaWgaaWcbaGaemyAaKgabeaakiab=jdiIkabcMcaPaWcbaGaemOAaOMaeyypa0JaeGymaedabaGafm4saSKbauaaa0GaeyyeIuoaaSqaaiabdMgaPjabg2da9iabigdaXaqaaiqbdUealzaafaaaniabggHiLdaakiaawIcacaGLPaaaaeaadaqadaqaaiabd6gaUnaaCaaaleqabaGaeGOmaidaaOGaeyOeI0IaemOBa4gacaGLOaGaayzkaaaaaaaacaWLjaGaaCzcamaabmaabaGaeGOnaydacaGLOaGaayzkaaaaaa@E72D@

*MDD*^*ind *^is then computed like *MDD *(expression 5), changing *RMM *elements by those of *RMM*^*ind*^. *RAR *is the correction of (1-*MDD*) for chance agreement and is the result of the following expression:

RAR=MDDind−MDDMDDind     (7)
 MathType@MTEF@5@5@+=feaafiart1ev1aaatCvAUfKttLearuWrP9MDH5MBPbIqV92AaeXatLxBI9gBaebbnrfifHhDYfgasaacH8akY=wiFfYdH8Gipec8Eeeu0xXdbba9frFj0=OqFfea0dXdd9vqai=hGuQ8kuc9pgc9s8qqaq=dirpe0xb9q8qiLsFr0=vr0=vr0dc8meaabaqaciaacaGaaeqabaqabeGadaaakeaacqWGsbGucqWGbbqqcqWGsbGucqGH9aqpdaWcaaqaaiabd2eanjabdseaejabdseaenaaCaaaleqabaGaemyAaKMaemOBa4MaemizaqgaaOGaeyOeI0Iaemyta0KaemiraqKaemiraqeabaGaemyta0KaemiraqKaemiraq0aaWbaaSqabeaacqWGPbqAcqWGUbGBcqWGKbazaaaaaOGaaCzcaiaaxMaadaqadaqaaiabiEda3aGaayjkaiaawMcaaaaa@483C@

Functions to compute the *RAR *measure for any two clusterings were implemented in MATLAB (Release 14), and are available in Additional file 4 or at the toolbox's webpage [22].

## Authors' contributions

FRP, MR and JSA conceived the study. FRP and JAC computationally implemented the new methods. FRP, MR and JAC interpreted the results. FRP wrote the manuscript. All authors revised and approved the final manuscript.

## Supplementary Material

Additional File 1 **Demonstration**. Pdf file with demonstration of *RAR *and *HA *equivalence in the absence of intercluster distance infromation. Click here for file

Additional File 2 **Comparison of simulated large clusterings**. Pdf file with methods, results and interpretation of the comparison of simulated large clusterings. Click here for file

Additional File 3 **Biological examples**. Pdf file with biological examples of the use of *RAR*. Click here for file

Additional File 4 **MATLAB toolbox**. Zip file with MATLAB functions to compute *RAR *and related measures. Click here for file
