# Supplementary material for: Ranked Adjusted Rand: integrating distance and partition information in a measure of clustering agreement
Source: BMC Bioinformatics. 2007 Feb 7;8:44. doi: 10.1186/1471-2105-8-44 (PMC1802093; doi:10.1186/1471-2105-8-44)
Supplement: Additional File 1 — Demonstration. Pdf file with demonstration of RAR and HA equivalence in the absence of intercluster distance infromation. [file 1471-2105-8-44-S1.pdf]

Suplement 1 - Equivalence of RAR and HA when distance information is not available for any of the two clusterings being compared

Both RMM and MM count entity pairs, but RMM counts the pairs *(i,j)* and *(j,i)* while MM counts only unique pairs. Consequently, the sum of all MM elements is $a+b+c+d=(n^2-n)/2$, while the sum of all RMM elements is (n^2-n). Independently of the availability of distance information, $rmm_{1,1}$ counts the number of entity pairs that are in the same cluster in both clusterings. This is also the kind of entity pairs that are counted in the element *a* of MM. As in RMM the order of the entities in the pair is accounted, $rmm_{1,1}$ is identical to $2a$. In the absence of distance information, the maximum intercluster distance rank is 1 and RMM is a 2 by 2 matrix. $rmm_{1,2}$ counts the pairs of entities that are in the same cluster in C and in different clusters in C' that have an intercluster distance rank of 1. It counts all the pairs that are in the same cluster in C and in different clusters in C', which is equivalent to $2b$. Analogously, $rmm_{2,1}$ is equal to the $2c$. Finally, $rmm_{2,2}$ counts the entity pairs that are in different clusters in both clusterings, which is identical to $2d$. Applying these four equivalences in expression (5) of the main text:

$$MDD_{nd} = \frac{2a\left|\frac{0}{1}-\frac{0}{1}\right| + 2b\left|\frac{0}{1}-\frac{1}{1}\right| + 2c\left|\frac{1}{1}-\frac{0}{1}\right| + 2d\left|\frac{1}{1}-\frac{1}{1}\right|}{n^2-n}$$

$$MDD_{nd} = \frac{b+c}{\left(n^2-n\right)/2} = \frac{b+c}{a+b+c+d} = 1-\frac{a+d}{a+b+c+d}$$

$$MDD_{nd} = 1-RI$$

Where RI is the Rand index and the index nd refers to the absence of intercluster distance information. Analogously:

$$MDD_{nd}^{ind} = \frac{rmm_{1,2}^{ind,nd} + rmm_{2,1}^{ind,nd}}{n^2-n} = 1-\frac{rmm_{1,1}^{ind,nd} + rmm_{2,2}^{ind,nd}}{n^2-n}$$

The (1,1) and (2,2) elements of RMM in the absence of can be determined through expression (6) of the main text:

$$rmm_{1,1}^{ind,nd} = \left(\sum_{i=1}^{K}(n_i^2-n_i)\right)\times\left(\sum_{i=1}^{K'}(n_i'^2-n_i')\right)\Big/(n^2-n) =$$

$$= \left(\sum_{i=1}^{K}(n_i^2)-n\right)\times\left(\sum_{i=1}^{K'}(n_i'^2)-n\right)\Big/(n^2-n) =$$

$$= \left(\sum_{i=1}^{K} (n_i^2) \sum_{i=1}^{K'} (n_i'^2) - n \left(\sum_{i=1}^{K} (n_i^2) + \sum_{i=1}^{K'} (n_i'^2) \right) + n^2 \right) / (n^2 - n) =$$

$$= \left(\sum_{i=1}^{K} \sum_{j=1}^{K'} (n_i^2 n_j'^2) - n \left(\sum_{i=1}^{K} (n_i^2) + \sum_{i=1}^{K'} (n_i'^2) \right) + n^2 \right) / (n(n-1)) =$$

$$= \left(\sum_{i=1}^{K} \sum_{j=1}^{K'} \left(\frac{n_i^2 n_j'^2}{n} \right) - \left(\sum_{i=1}^{K} (n_i^2) + \sum_{i=1}^{K'} (n_i'^2) \right) + n \right) / (n-1)$$

$$rmm_{2,2}^{ind,nd} = 2 \left(\sum_{i=1}^{K} \sum_{j=i+1}^{K} n_i \cdot n_j \right) \times 2 \left(\sum_{i=1}^{K'} \sum_{j=i+1}^{K'} n_i' \cdot n_j' \right) \Big/ (n^2 - n) =$$

$$= \left(\sum_{i=1}^{K} \sum_{j=1}^{K} n_i \cdot n_j - \sum_{i=1}^{K} n_i^2 \right) \times \left(\sum_{i=1}^{K'} \sum_{j=1}^{K'} n_i' \cdot n_j' - \sum_{i=1}^{K'} n_i'^2 \right) \Big/ (n^2 - n) =$$

$$= \left(\sum_{i=1}^{K} \left(n_i \cdot \sum_{j=1}^{K} n_j \right) - \sum_{i=1}^{K} n_i^2 \right) \times \left(\sum_{i=1}^{K'} \left(n_i' \cdot \sum_{j=1}^{K'} n_j' \right) - \sum_{i=1}^{K'} n_i'^2 \right) \Big/ (n^2 - n) =$$

$$= \left(\sum_{i=1}^{K} (n_i \cdot n) - \sum_{i=1}^{K} n_i^2 \right) \times \left(\sum_{i=1}^{K'} (n_i' \cdot n) - \sum_{i=1}^{K'} n_i'^2 \right) \Big/ (n^2 - n) =$$

$$= \left(n \sum_{i=1}^{K} (n_i) - \sum_{i=1}^{K} n_i^2 \right) \times \left(n \sum_{i=1}^{K'} (n_i') - \sum_{i=1}^{K'} n_i'^2 \right) \Big/ (n^2 - n) =$$

$$= \left(n^2 - \sum_{i=1}^{K} n_i^2 \right) \times \left(n^2 - \sum_{i=1}^{K'} n_i'^2 \right) \Big/ (n^2 - n) =$$

$$= \left(n^4 - n^2 \sum_{i=1}^{K} n_i^2 - n^2 \sum_{i=1}^{K'} n_i'^2 + \sum_{i=1}^{K} \sum_{j=1}^{K'} n_i^2 n_j'^2 \right) / (n(n-1)) =$$

$$= \left(n^3 - n \sum_{i=1}^{K} n_i^2 - n \sum_{i=1}^{K'} n_i'^2 + \sum_{i=1}^{K} \sum_{j=1}^{K'} \frac{n_i^2 n_j'^2}{n} \right) / (n-1)$$

The expected MDD for independent clusterings in the absence of intercluster distance information is then:

$$MDD_{nd}^{ind} = 1 - \frac{n + n^3 - (n+1) \sum_{i=1}^{K} n_i^2 - (n+1) \sum_{i=1}^{K'} n_i'^2 + 2 \sum_{i=1}^{K} \sum_{j=1}^{K'} \frac{n_i^2 n_j'^2}{n}}{n(n-1)^2}$$

$$MDD_{nd}^{ind} = 1 - \frac{\dfrac{n(n^2+1) - (n+1) \sum_{i=1}^{K} n_i^2 - (n+1) \sum_{i=1}^{K'} n_i'^2 + 2 \sum_{i=1}^{K} \sum_{j=1}^{K'} \frac{n_i^2 n_j'^2}{n}}{2(n-1)}}{n(n-1)/2}$$

$$MDD_{nd}^{ind} = 1 - \frac{n_c}{n(n-1)/2} = 1 - \frac{n_c}{a+b+c+d}$$

Knowing MDD_{nd} and MDD_{nd}^{ind}, RAR_{nd} can be deduced. By simplifying the expression of RARnd, it is possible to show that RAR in the absence of distance information is equivalent to the adjusted Rand index HA (expression (1) in the main text):

$$RAR_{nd} = \frac{MDD_{nd}^{ind} - MDD_{nd}}{MDD_{nd}^{ind}} =$$

$$RAR_{nd} = \frac{1 - \frac{n_c}{a+b+c+d} - \left(1 - \frac{a+d}{a+b+c+d}\right)}{1 - \frac{n_c}{a+b+c+d}} =$$

$$RAR_{nd} = \frac{-\frac{n_c}{a+b+c+d} + \frac{a+d}{a+b+c+d}}{\frac{a+b+c+d-n_c}{a+b+c+d}} =$$

$$RAR_{nd} = \frac{\frac{a+d-n_c}{a+b+c+d}}{\frac{a+b+c+d-n_c}{a+b+c+d}} = \frac{a+d-n_c}{a+b+c+d-n_c} = HA$$
