# Supplementary material for: Ranked Adjusted Rand: integrating distance and partition information in a measure of clustering agreement
Source: BMC Bioinformatics. 2007 Feb 7;8:44. doi: 10.1186/1471-2105-8-44 (PMC1802093; doi:10.1186/1471-2105-8-44)
Supplement: Additional File 2 — Comparison of simulated large clusterings. Pdf file with methods, results and interpretation of the comparison of simulated large clusterings. [file 1471-2105-8-44-S2.pdf]

Suplemment 2 - Analysis of large scale artificial clusterings

Simulation of clustering pairs

The generation of the pairs of clusterings to be compared is here described. Importantly, five factors were systematically varied:

1. Number of entities (100 to 2000)
2. Number of clusters (10 to 200)
3. Fraction of entities changing cluster membership and location (10% to 100%).
4. Ranked distance of the target cluster for entities changing cluster membership.
5. Distribution pattern of cluster sizes: from homogeneous clusters, clusters with random size, and clusterings with few big clusters and many small clusters.

The artificial clustering pairs were generated with the following procedure:

- An original cluster with a given number of entities (np), and a given number of clusters is generated:
    - Uniformly random coordinates for the cluster centroids are generated in a unit hypercube of 5 dimensions.
    - The first nc entities are attributed each to one of the nc clusters, so that every cluster has at least one element.
    - The nc+1 entity is attributed randomly to one of the nc clusters.
    - For every of the remaining entites, the corresponding cluster is attributed randomly, but each cluster has a probability of getting a new entity proportional to $e^{(\text{number of elements})*\text{alfa}}$. alfa is an input parameter that can vary from -0.05 to 0.05. When alfa=0, the cluster size distribution is random. When alfa is negative, the clusters tend to have all the same size. For growing positive values of alfa, the cluster size distribution approximates a power law, with few big clusters and many small clusters.
- The second clustering is initially generated as a copy of the first clustering.

- The number of clusters in the second clustering (nc2) is randomly chosen, varying from nc to 1.05*nc.
- If nc2>nc, coordinates for the new cluster centroids are randomly chosen.
- A fraction of moving entities (fme) is randomly chosen from the total number of entities.
- A ranked distance (rd) of the target cluster for moving entities is selected. For each selected moving entity, a cluster that is the rdth closest cluster to the original cluster of that entity is identified, and that entity is transferred from the original cluster to the new one.
- Ranked Adjusted Rand (RAR), Adjusted Rand (HA) and inter-entity correlation coefficient (r) are computed for the comparison of the two clusterings.

Partial linear correlation coefficients

To analyse the results of the simulated clustering comparisons, partial linear correlation coefficients were used. The partial correlation coefficient between two variables shows the strength of the linear relationship between both variables while the other variables under study are kept constant. In the present situation, three variables were being studied: RAR, HA and r. For any three variables x, y and z, the partial linear correlation coefficient between x and y keeping z constant is:

$$r_{x,y|z} = \frac{r_{x,y} - r_{x,z} \cdot r_{y,z}}{\sqrt{(1 - r_{x,z}^2) \cdot (1 - r_{x,y}^2)}}$$

In the previous expression, $r_{x,y}$ is the normal Pearson's linear correlation coefficient.

Analysis of simulation results

Computation time

The simulation studies showed that the number of clusters and the number of entities were the only two parameters influencing computation time. Of these parameters, the effect of the number of clusters shows a greater influence on computation time when compared with the number of entities. This result is shown on figure 1 of supplementary material. Computation time varies with the number of clusters

following a 4th degree polynomial, while for the number of entities it varies according to a 2nd degree polynomial. The effect of these two parameters on computation time is independent of each other. On a personal laptop (Intel® Pentium® M, 1.7 GHz, 512 MB RAM), the computation time for 1000 entities and 200 clusters was always less than 100 seconds.

RAR is robust to clustering factors

The only factor that had an effect on the final RAR values was the fraction of entities changing cluster membership and location, producing a linear correlation coefficient of r=-0.918. The number of entities (r=0.077), number of clusters (r=-0.109), ranked distance of the target cluster for entities changing cluster membership (r=-0.016) and the distribution pattern of entities (r=-0.032) had negligible impact on RAR values. Although we know that the ranked distance of the target cluster for entities changing cluster membership has a strong effect on RAR values, as seen with the small artificial example, its impact on the final spatial distribution of entities was unpredictable due to the way this parameter was implemented in the simulations. As the entities changing cluster membership were randomly selected from every possible cluster, the change in relative position of some entities could be balanced by entities moving in the opposite direction. Consequently, varying the extension of change in relative position produced highly variable results and a low correlation with RAR values (r=-0.016). Nevertheless, varying this factor was essential to generate clustering pairs with diverse inter-entity distance correlation coefficient (r). In fact, Suplementary Figure 2 shows a strong association between RAR and r values.

Weighting of partition and distance information

The relationships between RAR, HA and r are shown on the upper row of Suplementary Figure 2, where each point corresponds to a comparison between two simulated clusterings. The extensive variation of the fraction of entities changing cluster membership and location (10% to 100%). and the ranked distance of the target cluster for entities changing cluster membership, made it possible for the three measures to vary significantly. The three pair wise associations between each measure pair are strong and non-linear. The association that shows the highest strength is RAR-r. The weakness of the associations is here measured as the dispersion of the points around the major trend in the scatter plots of Suplementary Figure 2. The plots of RAR-HA and r-HA relationships show a considerable dispersion: the majority of the simulation points have an HA value around zero while RAR varies between 0.0

and 0.4 and r varies between 0.0 and 0.6. It is only for RAR values higher than 0.4, or r values higher than 0.6 that HA starts to correlate positively with these measures. When measure values are transformed in ranks, they approach linear relationships (lower row of Suplementary figure 2), allowing a partial correlation coefficient analysis (Suplementary Table 1). The partial correlation coefficient between two variables shows the strength of the linear relationship between both variables while the other variables under study are kept constant [22]. The partial correlation coefficient analysis can be used to avoid misinterpretation of results. For example, the fact that RAR-r is the strongest association added to a good correlation found between HA and r may suggest that the relationship between RAR and HA was due to the correlation that both measures have with r. This explanation is actually false as it can be shown by the analysis of Suplementary Table 1: the partial correlation coefficient between RAR and r (when HA is kept constant) and between RAR and HA (with r constant), are both strongly positive (0.76 and 0.72 respectively). This means that the co-variation between this pairs of variables is strong even in the absence of the effect of a third variable. In their whole, these results show that the RAR measurement encodes simultaneously two independent information sources: one related to the partition comparison (contained in HA) and another related with the maintenance of the relative distances between clusters (contained in r). Moreover, if RAR is kept constant, the partial-correlation coefficient between HA and r is slightly negative, corresponding to a weak correlation. This result strengths the hypothesis that it is RAR that is supporting the positive relationship between HA and r.

Suplementary Figures

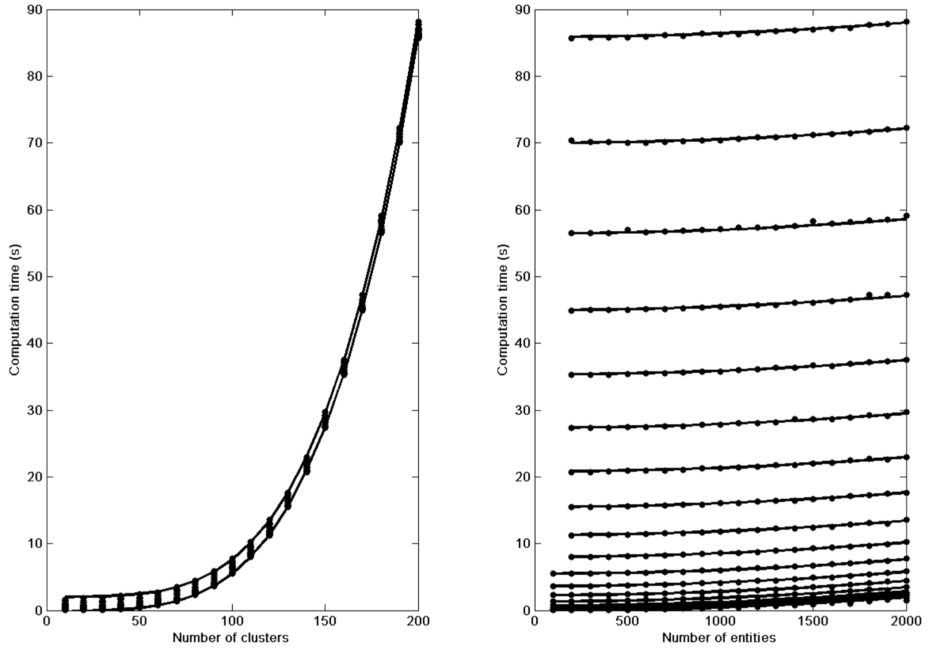

Supplementary figure 1 – Ranked Adjusted Rand (RAR) computation times for simulated clustering comparisons with different number of clusters and entities.

Each point corresponds to one clustering comparison with a fixed number of entities and of clusters (in both clusterings compared). The three factors: 1) fraction of moving entities, 2) ranked distance to target clusters of moving entities and 3) distribution pattern of cluster sizes were found to have no impact on computation time after previous tests, and for the presented plots were randomly varied. Lines represented in the figure are polynomial fits for subsets of plotted points. In the left figure, 4th degree polynomials achieved the best fit, while for the right figure 2nd degree polynomials gave optimal results. Choice of the polynomial degree that produced the best fit was made recurring to F tests.

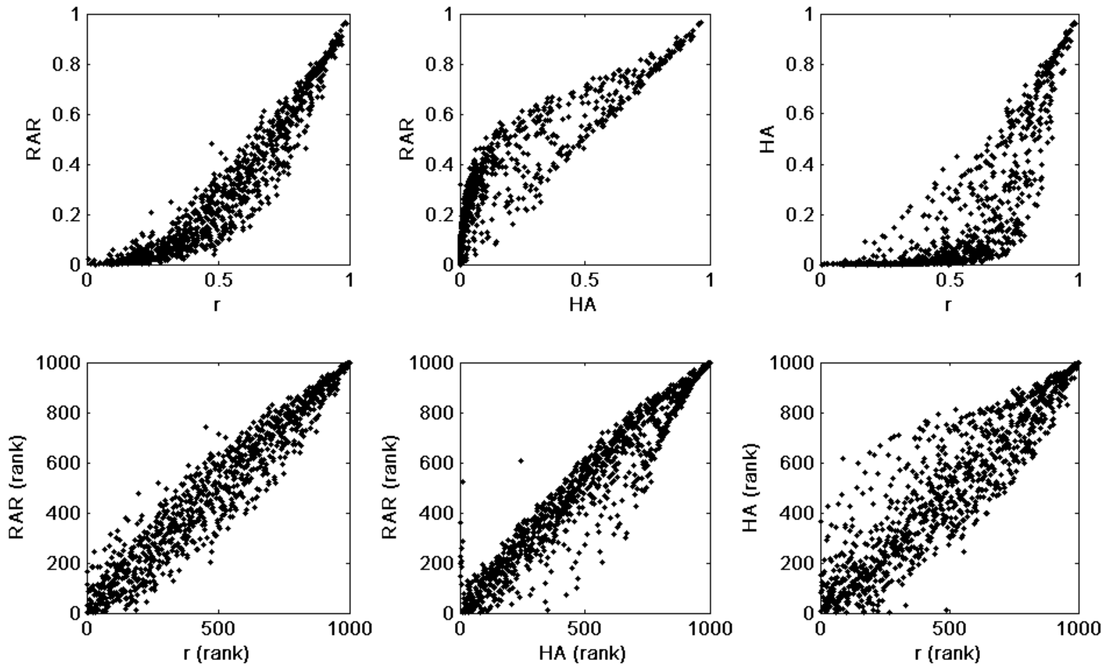

Supplementary figure 2 – Relationships between Ranked Adjusted Rand (RAR), Adjusted Rand (HA) and inter entity linear correlation coefficient (r).

The scatter plots in this figure were the result of 1000 simulated clustering comparisons. Each clustering randomly generated was compared with other obtained from the first by moving entities to different or new clusters. Five factors were randomly varied: number of entities (100-2000), number of clusters (10-200), fraction of entities changing cluster (0-100%), ranked distance of target cluster for moving entities (1-(number of clusters-1)), and distribution pattern of cluster sizes (a special parameter, varying from -0.05 to 0.05, was designed to change the cluster sizes from homogeneous clusters, through random sized cluster, until power-law like cluster size distributions. The referred parameter is described in supplementary material). The upper row shows scatter plots of the actual values of RAR, HA and R, while in the lower row the ranks of the values are plotted instead. Each point in the plots corresponds to one unique clustering comparison. Although the associations between the three measures are non-linear in their own scales, they are monotonically increasing, as shown by the linear relations when converted to ranks.

Supplementary Table 1– Comparison of RAR with inter-entities correlation coefficient (r) and with the Hubert's and Arabie's adjusted rand index (HA) applied to simulated clusterings.

All correlation coefficients were computed for a sample of 1000 simulated clustering comparisons. Calculus of partial linear correlation coefficient is described in supplementary material.

	Linear correlation coefficient between ranks		Partial linear correlation coefficient between ranks	
	r	HA	r	HA
HA	0.910		-0.134	
RAR	0.962	0.957	0.758	0.720
