# Supplementary material for: Ranked Adjusted Rand: integrating distance and partition information in a measure of clustering agreement
Source: BMC Bioinformatics. 2007 Feb 7;8:44. doi: 10.1186/1471-2105-8-44 (PMC1802093; doi:10.1186/1471-2105-8-44)
Supplement: Additional File 3 — Biological examples. Pdf file with biological examples of the use of RAR. [file 1471-2105-8-44-S3.pdf]

Supplement 3 - Analysis of biological data examples with RAR

Application 1 – comparing microbial typing methods

This example is described in the main text. This section of the supplement only contains an additional figure.

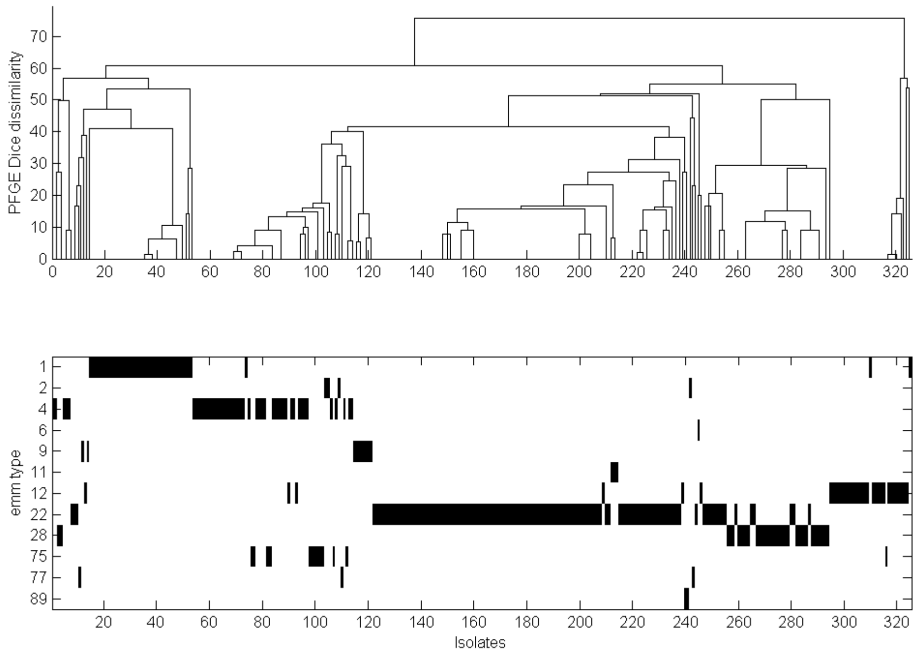

Figure 1. Microbial typing of 325 *S. pyogenes* isolates by PFGE and *emm* typing. Top plot: Dendrogram derived from PFGE band pattern comparisons using Dice dissimilarity and average linkage. Bottom plot: *emm* type of the same 325 isolates (isolates are ordered according to the leaf order of the dendrogram).

Application 2 – comparing regulatory network distance functions

This case study analyses the yeast's gene regulatory network organization. The directed graph model of this network was obtained from the work of Lee and colleagues with the experimental technique of chromatin immuno-precipitation on a chip [1]. The graph has 2415 nodes representing genes, 106 of them being also transcription factors. These 106 genes have outgoing interactions, connecting them to gene targets whose transcription they regulate. A possible approach to study this network organization is to cluster its nodes into consistent modules, but first a definition of the distance between nodes is needed. Here, the clusterings originated by two possible distance definitions are compared. The first definition is based upon the number of transcription factors that simultaneously and directly regulate a given pair of target genes (nodes). The greater the number of common transcription factors, the smaller is the distance between the nodes. The computed distance was the total number of

transcription factors in the network (106) subtracted by the number of shared factors). The second definition is to measure the distance between two nodes as the length of the shortest path connecting the nodes in the undirected version of the regulatory network. The hierarchical clustering algorithm with average linkage was applied for the two distance definitions [2]. For both clusterings 25 linkage distance threshold values were chosen, such that the resulting number of clusters would vary from 10 to 250, in equally spaced steps. Each cluster corresponds to a set of genes that are separated by a linkage distance equal or smaller that the linkage thresholds. For each of the 25 $\times$ 25 pairs of clusterings mean diagonal deviation (*MDD*), ranked adjusted rand (*RAR*), rand index (*R*), adjusted rand index (*HA*) and normalized variation of information (*VI*) were computed. Note that *RAR*, *R* and *HA* are agreement measures while *MDD* and *VI* are disagreement measures.

Figure 2 shows that, in this particular real data set, *RAR* and the *HA* produce similar results. An analogue parallel can be made between *MDD* and *VI*. Although correlations between these measures are clearly detected, they are not equivalent. The correlations reflect the fact that both *RAR* and *HA* (and *MDD* and *VI*) use the partition information to compare clusterings. But *MDD* and *RAR* also use information about the inter-cluster distances. It is this extra information that prevents the scatter plot of *RAR* versus *HA* (and also of *MDD* versus *VI*) from being a thin line.

In Figure 3 the visual representations of the *RMM* matrix are presented, one for the linkage threshold with the best agreement (according to *RAR*) and other for a representative sub-optimal agreement. These representations can enhance the comprehension of the level of agreement between the two clusterings. Comparing the two shown matrices it is easy to differentiate them. The left one has more red and yellow elements. These denser elements are more spread in the right matrix, thus corresponding to a weaker agreement. Indeed, the left matrix corresponds to the maximum *RAR* value observed for these clusterings at the different cluster numbers studied. In both matrices the more concentrated elements are not located exactly along the diagonal. Instead, they are slightly shifted upwards. This means that if two genes are members of close clusters according to the shortest path clustering, it is probable that they are located in even closer clusters (in terms of ranks) according to the number of common direct transcription factors. This kind of information is analogous to what is offered by asymmetric clustering comparison measures, but more detailed. There could be two clusterings *C* and *C'* where half of the clusters in *C* had a very good match in *C'*, but the other half of *C* clusters were split into the most distant clusters in *C'*. Due to the averaging effect, the *RAR* values could be similar to the comparison of clusterings *C* with *C''*, where all the clusters of *C* are split into not so distant clusters in *C''*, but the graphical representations of *RMM* for the *(C,C')* and *(C,C'')* comparisons would certainly be different. Computing the

mean diagonal deviations compresses the information contained in all the *RMM* matrix elements. In contrast with the previously available methods, *RAR* is inherently associated with this information rich visual output.

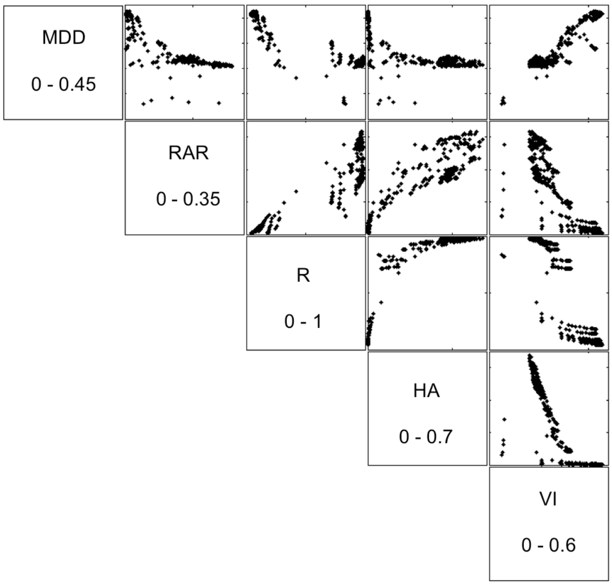

Figure 2. Multiple scatter-plots of comparison measures applied to clusterings of yeast's gene regulatory network by two distance definitions: 1) number of unshared direct transcription factors and 2) length of shortest path between the two nodes in the undirected graph of the regulatory network. Each subplot has 25 × 25 dots, one for each pair of clusterings with different linkage distance thresholds. In the diagonal squares the clustering comparison measures are identified, as well as the limits of the respective axes.

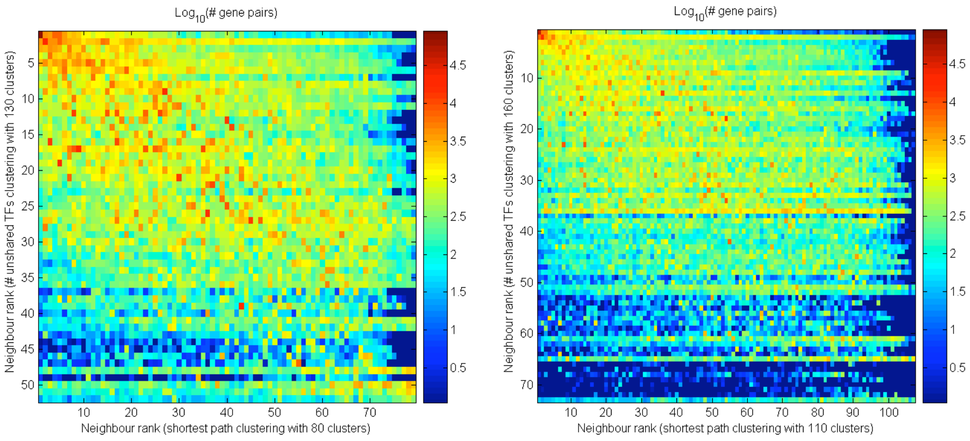

Figure 3. Graphical representations of *RMM*, the matrix that counts the entity pair (gene pair in this case) frequencies categorized by the neighbour rank between the respective clusters for both clusterings. If the two genes are in the same cluster according to the first (second) clustering they are going to contribute to the first row (column) of *RMM*. On the other extreme, if the two genes are in the most distant clusters according to the first (second) clustering they are going to contribute to the last row (column) of *RMM*. Each element of the matrix is here represented by a coloured rectangle. The colour codifies the frequency of gene pairs contributing to that element. The left matrix represents the best agreement (as measured by the maximum *RAR*) between the clusterings of the yeast's gene regulatory network using two distance definitions: the number of unshared direct transcription factors (y axis) and the shortest path in the undirected graph of the regulatory network (x axis).

Application 3 – comparing the agreement of different gene expression datasets with gene pathway information

The second case study used the comparison of clusterings to address the following question: does the quality of the perturbations applied in microarray experiments have an impact on the co-expression of functionally related genes? Three yeast gene expression data sets were obtained through the WebMiner web tool [3]. The first data set contained all the cell cycle related experiments available in WebMiner, comprising data from 77 arrays. The second data set contained all the experiments related with metabolism, comprising data from 11 arrays. Finally, the third set contained all the stress response experiments, corresponding to 19 arrays. The WebMiner tool also provided a pathway for some of the genes in the arrays (381 different pathway denominations where found for yeast genes). Only the 2512 genes involved in a known pathway (either experimentally confirmed or putative involvement) were used in this analysis. All the pair-wise correlations between expression profiles in each experiment set where computed. Correlation coefficients were subsequently transformed into correlation distances (1 – correlation coefficient) and used to hierarchically cluster the genes (again using the average linkage algorithm). As performed in the previous case study, 25 linkage distance thresholds were chosen, yielding 10 to 250 clusters. But now, all these different 3 (experiment type) $\times$ 25 clusterings were compared with the same gene pathway classification. For the pathway classifications there are no distances between clusters, since either two genes are involved in the same pathway or not. The same set of clustering comparison measures studied in the previous section was also applied here. To help in the interpretation of these results, the probability distributions of the pair-wise correlation distances, for gene pairs with the same pathway or not and for the three different data sets were estimated through kernel density estimation methods [4]. This case study provides a clear demonstration that the proposed measures can encode information that previous methods neglected. In Figure 4, both the *HA* and *VI* are unable to differentiate between the level of agreement (with gene pathway classification) obtained from metabolism related or stress response gene expression data. *R* is the least informative of all the tested measures, and equally evaluates the agreement between the three sets of gene expression data and gene pathway information. The inter-cluster distances in the gene expression data sets alone appeared sufficient to make *RAR* (and *MDD*) able to discriminate between the levels of agreement of the three kinds of experiments with the functional information about each gene. Additionally, the dissimilarity measures (*MDD* and *VI*), which are both uncorrected for chance agreement, are not concordant with the similarity measures (*RAR* and *HA*) about the agreement obtained with cell cycle gene expression data. The former consider cell cycle data as the closest to the pathway

classification but the latter, after the correction for the expected chance agreement, consider it the worst agreement (for *HA*) or the intermediate agreement (for *RAR*). Analysing the distributions of the correlation distances in Figure 5, the low performance of the cell cycle data for *RAR* and *HA* evaluations is justified, since the two distributions are very similar (which indicates that clusters containing pairs of genes involved in the same pathway may easily have also genes involved in different pathways because the pair-wise distances have similar ranges).

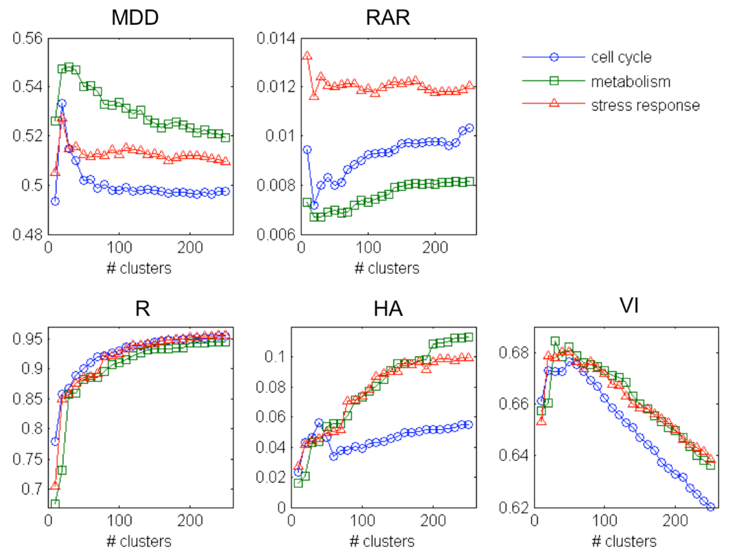

Figure 4. Quantitative evaluation of the agreement between gene expression clusterings and gene pathway classification through five different measures (one in each subplot) for different gene expression cluster numbers (x axis) and for three different types of experiments (different line colours and markers).

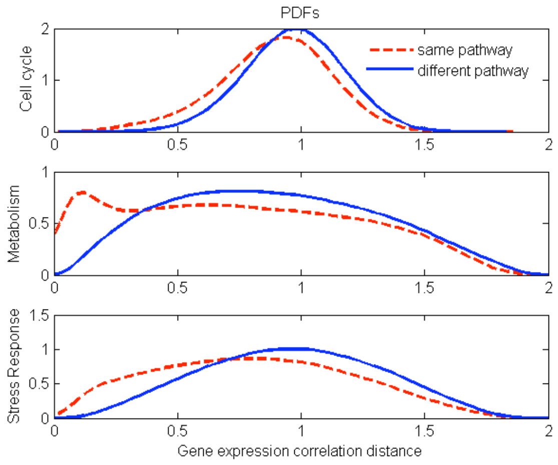

Figure 5. Probability density functions of the pair-wise correlation distances, for gene pairs involved in the same pathway (red interrupted line) or not (blue continuous line) and for the three different gene expression data sets (one in each subplot).

The data set of stress response experiments originates a larger difference between the two distributions, such that genes involved in different pathways tend to have higher correlation distances between expression profiles. This is reflected in the high *RAR* and *HA* for the agreement of stress response gene expression data clustering and functional annotation. For metabolism related experiments, there is a stronger probability that genes involved in different pathways have a relatively low correlation distance (in figure 5, the blue curve maximum is shifted to the left, as compared with cell cycle and stress response experiments). This is the reason why the worst agreements according to *RAR* are achieved with metabolism related data. HA is not sensitive to this distribution shift, even considering that the number of gene pairs involved in different pathways is much larger, and, consequently, changes in the distribution of correlation distances for genes involved in different pathways should have a major impact in clustering comparison measures.
This example shows that RAR has a higher discriminatory power, as compared with HA, R and VI, and that it is the only measure that is sensitive to differential expression disagreement of pair of genes involved in different pathways.

1. Lee TI, Rinaldi NJ, Robert F, Odom DT, Bar-Joseph Z, Gerber GK, Hannett NM, Harbison CT, Thompson CM, Simon I, et al: **Transcriptional regulatory networks in Saccharomyces cerevisiae.** *Science* 2002, **298:**799-804.
2. Sneath PH, Sokal, R. R.: *Numerical Taxonomy.* San Francisco: Freeman; 1973.
3. Heiman MG, Walter P: **Prm1p, a pheromone-regulated multispanning membrane protein, facilitates plasma membrane fusion during yeast mating.** *J Cell Biol* 2000, **151:**719-730.
4. Quinn GP, Keough, M. J.: *Experimental design and data analysis for biologists.* Cambridge: Cambridge University Press; 2002.
